# DNA Damage Repair Gene Set as a Potential Biomarker for Stratifying Patients with High Tumor Mutational Burden

**DOI:** 10.3390/biology10060528

**Published:** 2021-06-14

**Authors:** To-Yuan Chiu, Ryan Weihsiang Lin, Chien-Jung Huang, Da-Wei Yeh, Yu-Chao Wang

**Affiliations:** 1 Institute of Biomedical Informatics, National Yang Ming Chiao Tung University, Taipei 112304, Taiwan; chuedelilah@ym.edu.tw (T.-Y.C.); ryanlin0828@berkeley.edu (R.W.L.); gummy.md09@nycu.edu.tw (C.-J.H.); toh82228@ym.edu.tw (D.-W.Y.); 2 Center for Systems and Synthetic Biology, National Yang Ming Chiao Tung University, Taipei 112304, Taiwan

**Keywords:** tumor mutational burden, DNA damage repair genes, immunotherapy, biomarker, biomedical informatics

## Abstract

**Simple Summary:**

Immunotherapy has been a promising therapeutic approach for cancer treatment in recent years. Although cancer immunotherapy has achieved remarkable success, treatment response is only observed in a small number of patients. As nonresponders need to endure high treatment costs and toxicities with little benefit from treatment, identifying potential predictive biomarkers is critical to optimize the benefits of immunotherapy in patients. The total number of mutations in the tumor genome is a useful biomarker. Patients with a large number of mutations tend to respond better to cancer immunotherapy. However, assessment of the total number of mutations may not be easy. In this study, we identified gene sets with only a small number of genes whose mutations serve as an indicator of the total number of mutations. These cancer-specific gene sets can be used as a cost-effective approach to stratify patients with a large number of mutations in clinical practice.

**Abstract:**

Tumor mutational burden (TMB) is a promising predictive biomarker for cancer immunotherapy. Patients with a high TMB have better responses to immune checkpoint inhibitors. Currently, the gold standard for determining TMB is whole-exome sequencing (WES). However, high cost, long turnaround time, infrastructure requirements, and bioinformatics demands have prevented WES from being implemented in routine clinical practice. Panel-sequencing-based estimates of TMB have gradually replaced WES TMB; however, panel design biases could lead to overestimation of TMB. To stratify TMB-high patients better without sequencing all genes and avoid overestimating TMB, we focused on DNA damage repair (DDR) genes, in which dysfunction may increase somatic mutation rates. We extensively explored the association between the mutation status of DDR genes and TMB in different cancer types. By analyzing the mutation data from The Cancer Genome Atlas, which includes information for 33 different cancer types, we observed no single DDR gene/pathway in which mutation status was significantly associated with high TMB across all 33 cancer types. Therefore, a computational algorithm was proposed to identify a cancer-specific gene set as a surrogate for stratifying patients with high TMB in each cancer. We applied our algorithm to skin cutaneous melanoma and lung adenocarcinoma, demonstrating that the mutation status of the identified cancer-specific DDR gene sets, which included only 9 and 14 genes, respectively, was significantly associated with TMB. The cancer-specific DDR gene set can be used as a cost-effective approach to stratify patients with high TMB in clinical practice.

## 1. Introduction

In recent years, immune checkpoint inhibitors (ICIs) have resulted in good clinical responses to different cancers. Two main types of immune checkpoints are currently used for anticancer drugs: cytotoxic T-lymphocyte-associated antigen 4 (CTLA-4) and programmed cell death 1 (PD-1). However, ICIs still have limitations in cancer therapy. Currently, only a few patients benefit from ICIs [1]. As ICI therapy is costly, it is necessary to screen biomarkers for immunotherapy to better stratify patients and identify those who may respond well to treatments.

Tumor mutational burden (TMB) is defined as the total number of mutations in the tumor genome. The assessment of TMB as a clinical predictive biomarker for immunotherapy has increased awareness in recent years. A significant association between high TMB and ICI response has been reported in many studies for different tumor types, including non-small cell lung cancer (NSCLC), melanoma, and urothelial carcinoma [2,3]. As high somatic variants may generate more neoantigens, some of which would facilitate immune recognition as tumor foreignness and increase neoantigen-reactive T cells with antitumor immune responses, patients treated with ICIs have a better response [4].

Although TMB seems to be a promising predictive biomarker for cancer immunotherapy, some limitations still hinder its clinical application. The first is the method used to assess the TMB. Currently, the gold standard for calculating TMB is whole-exome sequencing (WES) [5]. However, high cost, long turnaround time, infrastructure requirements, and bioinformatics demands have prevented WES from being employed in routine clinical practice. The development of targeted next-generation sequencing (NGS) panels, which can accurately estimate TMB, has been advocated as a predigested and cheaper method [6]. For instance, comprehensive genomic profiling (CGP) assay can demonstrate good performance in TMB assessment [7]. Recently, the FDA approved pembrolizumab for adults and children with TMB-high solid tumors, which is defined as ≥10 mutations per megabase assessed using the FoundationOneCDx assay.

Although panel-sequencing-based estimates of TMB have gradually replaced WES TMB in clinical practice [8], scientists are still exploring various methods to fine-tune panel-based TMB as inconsistencies between panel-based TMB and WES TMB have been observed in various studies [7,9,10,11]. This inconsistency might result from panel design biases, which leads to the overestimation of TMB [12]. Moreover, as TMB distribution varies among different cancer types, it may not be appropriate to use a fixed TMB threshold to identify TMB-high patients across different tumors [13,14]. Therefore, the indeterminacy of the TMB threshold to classify patients with high/low TMB should be considered. To avoid overestimating TMB and to identify patients with high TMB better, our goal is to design a panel that allows us to focus specifically on distinguishing TMB-high patients from others. This will help to simplify the TMB evaluation process in clinical practice.

DNA damage repair (DDR) is the detection of alterations in DNA chemical structure and correction of alterations [15]. In human cells, both metabolic activities and radiation can lead to DNA damage and mutations. When damage cannot be restored, cell death occurs. Consequently, defects in DDR genes may lead to genomic instability and cancer risk [16,17]. DDR deficiency has also been observed to play important roles in cancer metastasis [18]. The association between a higher TMB and DDR deficiency has been reported in many studies [19,20,21]. In the human genome, 276 genes were identified as DDR genes. These 276 DDR genes can be classified into 9 DDR pathways: base excision repair (BER), nucleotide excision repair (NER), mismatch repair (MMR), Fanconi anemia (FA) pathway, homology-dependent recombination (HR), nonhomologous DNA end joining (NHEJ), direct damage reversal/repair (DR), translesion DNA synthesis (TLS), and other pathways [16]. Distinct DNA damage-related mutational signatures have been detected in different DDR pathways [22]. Previous studies have shown that DDR pathway disorders may increase TMB in tumors. Specifically, mutations in MMR genes (*MLH1*, *MSH2*, *MSH6*, and *PMS2*) lead to the loss of DDR activity and contribute to hypermutation in colorectal cancer and stomach adenocarcinoma [23,24]. Hence, variations in DDR genes are more likely to be correlated with high TMB and have better clinical outcomes with ICIs.

A previous study reported that mutations in MMR and polymerase (POL) genes (including *POLD1* and *POLE*) are associated with high TMB [23]. A cohort of 2885 pediatric tumors, including three tumor types (malignant gliomas, colorectal cancers, and leukemia/lymphomas) were used to investigate the association between TMB and the mutation status of MMR and POL genes. Combined defects in MMR and POL genes represent significant hypermutation, while defects in only POL genes do not represent hypermutation [23]. Another study also showed that the mutation status of MMR genes and the *POLE* gene is associated with high TMB [7]. They used a cohort of 92,438 tumors obtained from over 100 different cancer types and showed that the mutation of MMR genes or *POLE* gene would result in higher TMB in a pan-cancer scenario.

From these studies, we found that these studies were confined to only MMR and POL genes rather than all DDR genes. Further, a pan-cancer analysis was conducted instead of investigating different cancer types individually. Therefore, we aimed to clarify the relationship between the mutation status of DDR genes and TMB in various cancer types. Knijnenburg et al. gathered 80 DDR core genes from 276 DDR genes, which are the essential repair genes from different types of DNA damage [16]. Therefore, we sought to construct a panel that contains a specific gene set that detects TMB-high patients for different cancers from these 80 DDR core genes. This panel can be used as a cost-effective approach to stratify patients with high TMB in clinical practice.

## 2. Materials and Methods

### 2.1. Mutation Data

In this study, the mutation data were obtained from The Cancer Genome Atlas (TCGA) multicenter mutation calling in multiple cancers (MC3) project [25], which includes 10,294 tumor samples and 21,320 genes across 33 different cancer types. As the variant annotation of the MC3 data was called at least twice from the seven variant calling algorithms, the high-confidence somatic mutations were used for further analysis. Primary solid tumor samples were selected for our analysis, except for acute myeloid leukemia, in which primary blood-derived samples were selected.

### 2.2. Mutation Matrix Construction

Using the TCGA MC3 mutation data, a mutation matrix and a nonsynonymous mutation matrix for each cancer type were constructed. Nine of the sixteen categories of “Variant_Classification”, including missense mutation, frameshift del, splice site, nonsense mutation, frameshift ins, in-frame del, in-frame ins, nonstop mutation, and translation start site, were considered as nonsynonymous mutations. Moreover, to ensure that the variants would have a potential impact on the translated proteins, we screened the “IMPACT” column, which indicates the predicted variant effects. Samples with high, low, and moderate impacts were preserved for nonsynonymous mutation matrix construction. In the nonsynonymous mutation matrix, each row indicates a gene, and each column represents a patient. Each element in the matrix reveals the number of nonsynonymous mutations in a specific gene in a particular patient. A mutation matrix that included both synonymous and nonsynonymous mutations was also constructed. The column sum in the mutation matrix was considered as the TMB for a particular patient.

### 2.3. Examination of the Association between the Mutation Status of DDR Genes and TMB

For each DDR gene, patients were divided into two groups based on the mutation status of the gene: the mutated group (nonsynonymous variant) and the wild-type group. The Mann–Whitney U test was used to investigate whether a significant difference existed between the TMBs of the two groups. A significant difference (*p* < 0.05) indicated that the mutation status of the DDR gene was significantly associated with TMB.

### 2.4. Identification of a DDR Gene Set as a Potential Biomarker

To identify a DDR gene set as a potential biomarker for stratifying patients with high TMB, we calculated the effect sizes and the corresponding standard scores for each gene set. Subsequently, we used a stepwise selection method to identify the optimal gene set with a maximal standard score.

#### 2.4.1. Effect Size Calculation

Cohen’s *d* was applied to measure effect size, which indicates the standardized difference between the means of measurements [26]. Here, we compared the TMB difference between the mutated and wild-type groups. The formula for Cohen’s *d* is

(1) $$d=\frac{M_{\mathrm{mutated}}-M_{\mathrm{wild}\text{-}\mathrm{type}}}{\sqrt{\frac{SD_{\mathrm{mutated}}^{2}+SD_{\mathrm{wild}\text{-}\mathrm{type}}^{2}}{2}}}$$

where *d* is the result of Cohen’s *d*, showing the effect size of the difference between the two groups, *M*_mutated_ denotes mean TMB of the mutated group, *M*_wild-type_ denotes mean TMB of the wild-type group, and *SD*_mutated_ and *SD*_wild-type_ indicate standard deviations for the mutated and wild-type groups, respectively. Notably, the sign of Cohen’s *d* was important in our study. A positive effect size meant that the average TMB of the mutated group was higher than that of the wild-type group, whereas a negative effect size indicated a higher average TMB of the wild-type group. Therefore, we evaluated the degree of difference between the mutated and wild-type groups in terms of the effect size of the DDR gene set in our study.

As the calculation of effect size may be affected by the number of genes in the gene set and our aim was to identify an optimal gene set as a potential biomarker, the calculated effect sizes need to be transformed for comparison. Here, the concept of a standard Z-score was adopted. The standard score was computed as the degree of standard deviation from the mean of the effect size distribution consisting of the calculated effect sizes for the same number of genes in the gene set. A positive score indicates that the calculated effect size is larger than the mean effect size of the distribution, and vice versa. The effect size distribution was constructed either using all the calculated effect sizes or 10,000 random samplings of gene sets and their corresponding effect sizes.

#### 2.4.2. Stepwise Selection Method

One of the challenges is identifying the best set of genes. Tremendous numbers of possible combinations forbid us to explore all possibilities fully. Therefore, based on the effect size and the corresponding standard score calculated for each gene set, the stepwise selection method, which combines the forward selection method with the backward elimination method, was applied to identify the optimal gene set. The detailed gene set selection process is illustrated in Figure S1.

## 3. Results

### 3.1. Association between the Mutation Status of the DDR Genes and TMB

To investigate the association between the mutation status of the DDR genes and TMB, we downloaded the mutation data in the TCGA MC3 project and constructed the mutation matrices for 33 cancer types. Based on the constructed nonsynonymous mutation matrices, the samples were divided into two groups, mutated and wild-type, according to the mutation status of the DDR genes. The Mann–Whitney U test was employed to inspect the association between TMB and the mutation status of the DDR genes.

**Figure 1:**
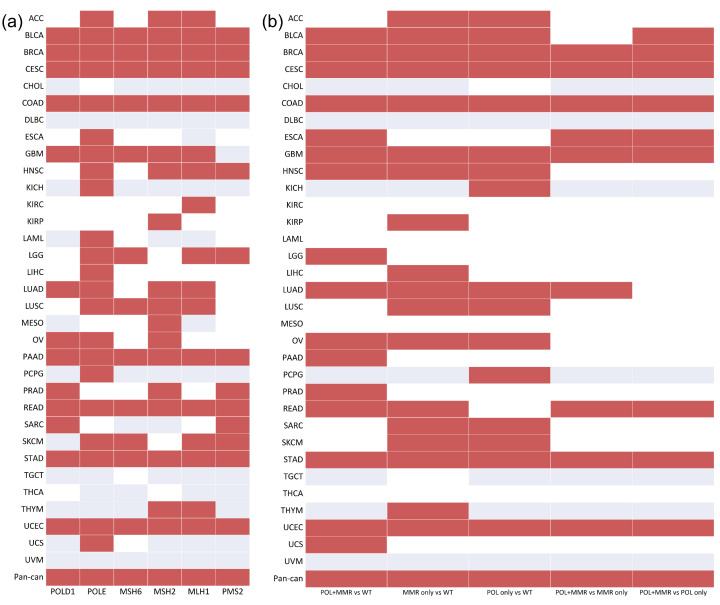
The association between TMB and the mutation status of the MMR and POL genes/gene sets in 33 cancer types and pan-cancer: (**a**) MMR and POL genes; (**b**) MMR and POL gene sets. Red indicates that the mutation status of the specific gene/gene set is significantly associated with high TMB. White indicates that mutation in the specific gene/gene set has no significant association with high TMB. Gray indicates that there is no mutation in this specific gene/gene set.

As a previous study reported that mutations in MMR (*MSH2*, *MLH1*, *MSH6*, and *PMS2*) and POL (*POLD1*, *POLE*) genes are associated with high TMB [23], we initially investigated the association between TMB and the mutation status of MMR and POL genes in 33 cancer types and pan-cancer. No single MMR or POL gene with mutation status was significantly associated with high TMB in all 33 cancer types and pan-cancer (Figure 1a). However, a significant association between the mutation status of *POLE* and high TMB was observed in 22 cancer types.

From the perspective of the gene set, the association between the mutation status of MMR, POL, and POL + MMR (at least one mutation in both gene sets) was investigated. In breast invasive carcinoma (BRCA), cervical squamous cell carcinoma and endocervical adenocarcinoma (CESC), colon adenocarcinoma (COAD), esophageal carcinoma (ESCA), glioblastoma multiforme (GBM), rectum adenocarcinoma (READ), stomach adenocarcinoma (STAD), and uterine corpus endometrial carcinoma (UCES), tumors with a combined MMR deficiency and polymerase mutation displayed a significant hypermutant phenotype than tumors with mutations in the MMR or POL gene sets only. Furthermore, gene set mutation status (MMR, POL, and MMR + POL) was significantly associated with high TMB in over half of the cancer types (Figure 1b).

We then analyzed 276 DDR genes to acquire specific genes in which the mutation status was significantly associated with high TMB. The results showed that the mutation status of most DDR genes was significantly associated with high TMB in various cancers. In pan-cancer, the mutation status of the DDR genes was significantly associated with high TMB, excluding the *IDH1* gene (Figure S2).

**Figure 2:**
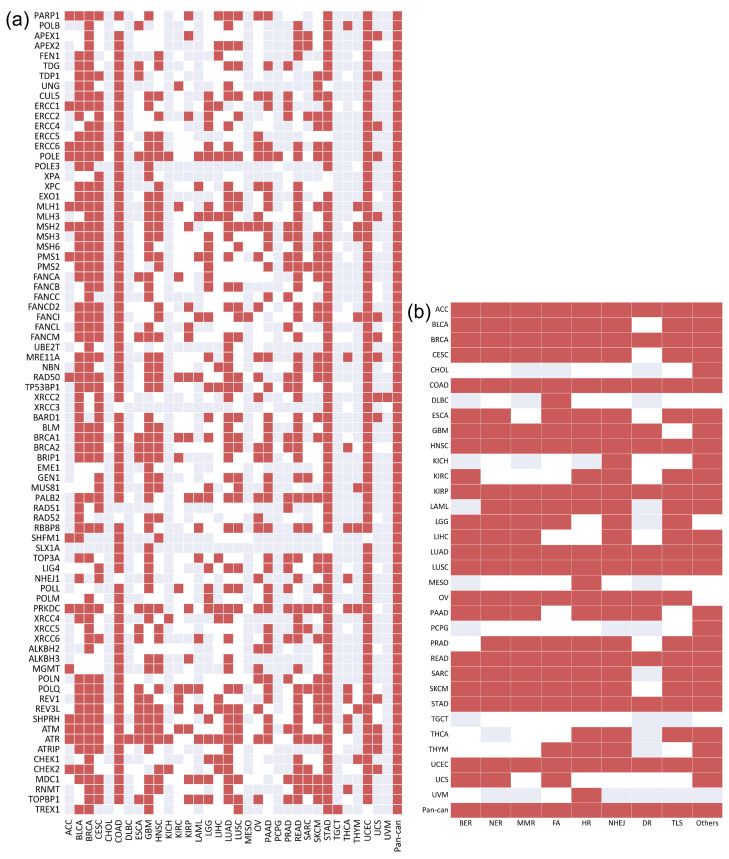
The association between TMB and the mutation status of the 80 DDR core genes/9 core DDR pathways in 33 cancer types and pan-cancer: (**a**) shows 80 DDR core genes. The density of the significant DDR core genes in the heatmap is approximately 0.6053; (**b**) shows 9 core DDR pathways. The density of the significant core DDR pathways is approximately 0.7645. Red indicates that the mutation status of the specific gene/DDR pathway is significantly associated with high TMB. White indicates that mutation in the specific gene/DDR pathway has no significant association with high TMB. Gray indicates that there is no mutation in this specific gene/DDR pathway.

The significant association of the 80 DDR core genes gathered by Knijnenburg et al. [16] is shown in Figure 2a. Correspondingly, the mutation status of most DDR core genes was found to be significantly associated with high TMB in multiple cancers. In pan-cancer, the mutation status of all DDR core genes was significantly associated with high TMB. Further, the density of the 80 DDR core genes was higher than that of significant DDR genes (0.6053 vs. 0.5671), indicating that the mutation status of these selected DDR core genes may be more associated with high TMB.

These 80 DDR core genes can be classified into nine core DDR pathways, which are applied to repair different damage types. We regarded each pathway as a specific DDR core gene set. Neither pathway was found to be significantly associated with high TMB across all 33 cancer types and pan-cancer (Figure 2b). However, the density of the DDR pathway was substantially higher than that of the DDR core genes (0.7645 vs. 0.6053), indicating that the mutation status of the gene sets may be a more appropriate indicator of high TMB than individual genes.

### 3.2. Identification of a DDR Gene Set as a Potential Biomarker

To find a DDR gene set from these 80 DDR core genes, the mutation status of which is significantly associated with high TMB in each cancer, we calculated the effect sizes of specific gene sets and subsequently transformed each effect size into a standard score based on the cancer type specificity. A stepwise selection method was then used to identify the optimal gene set for each cancer. As immunotherapies are often used to treat patients and exhibit a better response in skin cutaneous melanoma (SKCM) [27,28] and lung adenocarcinoma (LUAD) [29,30], we identified cancer-specific gene sets in these cancers. The optimal gene sets identified for SKCM and LUAD are listed in Table 1.

**Figure 3:**
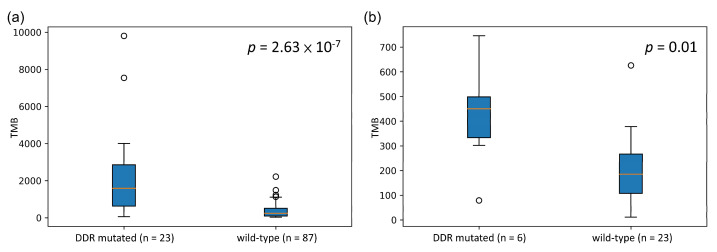
Boxplot of TMB distribution in both the mutated and wild-type groups for SKCM and LUAD: (**a**) SKCM and (**b**) LUAD. *p*-value was calculated by the Mann–Whitney U test.

**Figure 4:**
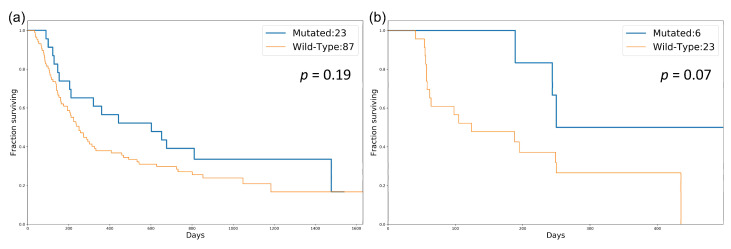
Survival analysis for patients in the mutated and wild-type groups in SKCM and LUAD: (**a**) comparison of OS in SKCM; (**b**) comparison of PFS in LUAD. *p*-value was calculated by the log-rank test.

We sought to validate whether our identified cancer-specific gene set could be a potential biomarker for stratifying patients with high TMB. Therefore, some independent datasets with both mutation data and treatment response information were retrieved for validation [31,32]. Based on the mutation status of the identified gene set, patients were separated into two groups (DDR-mutated and wild-type). The TMBs of these two groups were compared to determine whether there was a significant difference. The boxplots representing the association between the mutation status of the identified gene set and TMB for both SKCM and LUAD are shown in Figure 3. These validation data showed that the mutation status of these identified gene sets is significantly associated with high TMB in both SKCM and LUAD. Subsequently, we performed a survival analysis to compare the overall survival (OS) and progression-free survival (PFS) among patients in the mutated and wild-type groups (Figure 4). OS events in SKCM and PFS in LUAD events were obtained from Van Allen et al. [32] and Rizvi et al. [31], respectively. The survival analysis results showed that the mutated group in both SKCM and LUAD tended to have better survival. However, these differences were not statistically significant.

## 4. Discussion

In recent years, immunotherapy has become a promising cancer therapy. Many studies have shown that TMB is a potential predictive biomarker for cancer immunotherapy, indicating that patients with high TMB have better responses to ICIs [19]. In addition, many studies have shown that alterations in DDR pathways may contribute to increasing TMB in tumors. Specifically, mutations in MMR genes have been demonstrated to result in defective DDR pathways and promote hypermutation in colorectal cancer [23] and stomach adenocarcinoma [24]. However, the association between TMB and mutation status in DDR has not been extensively investigated. Therefore, the first goal of our study was to clarify the association between the mutation status of DDR genes and TMB across all 33 cancer types in the TCGA database. We found no single DDR gene/pathway with mutation status that was significantly associated with high TMB in all 33 cancer types. In some cancers, combined defects in the MMR and POL gene sets display significant hypermutation, compared to defects in only MMR or POL gene sets. In addition, in pan-cancer, the mutation status of any DDR core gene was significantly associated with high TMB.

According to our analysis, no DDR gene or pathway can be utilized to identify patients with high TMB across all 33 cancer types. To solve this problem, based on cancer-type specificity, we employed a stepwise selection method to find a cancer-specific gene set for each cancer. We identified gene sets for both SKCM and LUAD because patients with these cancers often receive immunotherapies and have better outcomes. To validate our identified gene sets in SKCM and LUAD, we used independent data to determine whether the identified gene sets could be used to stratify patients with high TMB or better response to immunotherapy. Consequently, the mutation of our identified gene set in each cancer could indeed contribute to the high TMB. Furthermore, the results of the survival analysis showed that the mutated group tended to respond better; however, the differences were not significant in either SKCM or LUAD.

In the present study, we attempted to interpret the biological implications of our identified gene sets in SKCM and LUAD. In SKCM, we found that most of the identified genes are the core genes of MMR and HR pathways, which participate in the repair of DNA damage during DNA replication [33,34]. We hypothesized that dysfunction in the MMR and HR pathways is associated with high TMB. SKCM has a higher frequency of DNA duplication and cell division [35], which may increase the need for corrections in alterations. In general, the MMR and HR pathway genes can detect DNA damage during repair. However, when the damage cannot be revived, patients with SKCM would have higher TMB. Thus, we believe that mutations in the MMR and HR pathways would contribute to high TMB. In contrast, we found that the genes in our identified gene set in LUAD had functions across different DDR-related pathways. These pathways may act alone or together to repair DNA damage. Accordingly, we believe that the cause of the high TMB in patients with LUAD is complicated.

We focused on the alteration in DDR genes rather than in all genes. Therefore, we sought to clarify whether the mutation status of the non-DDR gene set is more associated with TMB than the identified DDR gene set in SKCM and LUAD. Simple random sampling was performed to compare the effect sizes from the non-DDR gene sets with those from the identified DDR gene set (Figure S3). The remaining DDR gene sets (excluding our identified set) were also compared with the identified DDR gene set (Figure S4). The results of these random samplings showed that the mutation status of the identified DDR gene set in SKCM/LUAD was more significantly associated with high TMB than most gene sets of the non-DDR and the remaining DDR. Due to computational complexity, we used the stepwise selection method instead of running all combinations to identify the optimal gene set. Consequently, we cannot guarantee that the identified gene set in our study was indeed the best of all the possible combinations. Nevertheless, we revealed that the identified DDR gene set is a meaningful gene set that outperforms most randomly selected gene sets in SKCM and LUAD.

As SKCM and LUAD are generally characterized by high TMB, which may be caused by strong carcinogens such as UV light from the sun, cigarette smoking, or air pollution, we also applied our method to COAD, which is not mainly caused by strong carcinogens. We could identify a specific gene set that was significantly associated with high TMB based on the TCGA COAD mutation data (Figure S5a). With the independent dataset used for validation [36] and the identified COAD gene set, the DDR-mutated patients had significantly higher TMB than the wild-type patients (Figure S5b). Moreover, the results of the random sampling indicated that the identified COAD gene set outperformed the other gene sets of the same size (Figure S5c,d). Based on the results of SKCM, LUAD, and COAD, we believe that the proposed computational algorithm can also be used in other cancer types.

Targeted gene panels, such as FoundationOne CDx and others, are widely used to determine TMB. However, the TMB threshold to classify patients into TMB-high or TMB-low is still undetermined. A research study had shown that the misclassification rate may vary from 30% to <1% between the cutoff of 5 to 40 mutations per Mb for those commonly used panels. With the cutoff of 10 mutations per Mb, the most frequently used threshold of high TMB, the misclassification rates are still 4% to 10% for different panels [37]. Therefore, the goal of this research was aimed at designing a method that could bypass the step of determining the TMB cutoff threshold while still being able to stratify patients with high TMB. According to the mutation status of the identified gene sets, patients can be classified as DDR-mutated or wild-type. Those patients with mutated DDR gene set were recognized as patients with high TMB, thus bypassing the determination of TMB threshold in different cancer types. Here, we compared the identified DDR gene sets and FoundationOne CDx panel to verify the capability of stratifying TMB-high patients. In addition, 10 mutations per Mb was used as the threshold for FoundationOne CDx as FDA approved. The identified DDR gene sets had a higher precision to determine TMB-high patients, compared to FoundationOne CDx in SKCM, LUAD, and COAD, using WES TMB as the reference (Figure S6a). In fact, based on the design of the proposed methods, the TMB-high patients identified by our DDR gene sets would be those with ultra-hypermutation. Therefore, we further examined the TMB amplitude between the TMB-high patients identified by our gene sets and those by the FoundationOne CDx panel. The results indicated that the TMB of the patients identified by the DDR gene sets is much higher in all three cancer types (Figure S6b), demonstrating that our method indeed identified ultra-hypermutant tumors as we expected. Ultra-hypermutant tumors were highly associated with germline cancer predisposition involving replication repair genes. Therefore, ultra-hypermutant is used as a biomarker in the screening processing for early tumor detection [23]. Consequently, we believe that the identified DDR gene sets could potentially provide preclinical benefits since they can use a relatively small size of genes to stratify ultra-hypermutants.

The use of TMB as a potential biomarker for immunotherapy response has various limitations in clinical use as its assessment by WES or targeted NGS panel has a high cost, long turnaround time, infrastructure requirements, and bioinformatics demands. Even if the targeted NGS panel is cheaper than WES, defining the TMB threshold to stratify patients with high TMB remains challenging. In our study, because we stratified patients based on the mutation status of a specific gene set, patients can be simply divided into either mutated or wild-type groups without determining the TMB threshold for TMB-high patients. Therefore, patients with mutations in a specific gene set are predicted to be responders to cancer immunotherapy. More independent data are needed to test the predictive performance of the identified cancer-specific gene sets.

In conclusion, we investigated the association between the mutation status of DDR genes and TMB. Additionally, we proposed a computational algorithm to identify a cancer-specific gene set that can be used to stratify patients with high TMB, particularly ultra-hypermutant patients. We applied our algorithm to SKCM and LUAD and demonstrated that the mutation status of the identified cancer-specific DDR gene set was significantly associated with TMB. We expect that the identified cancer-specific DDR gene set can be used as a biomarker for immunotherapy response prediction in clinical practice and for preclinical early tumor detection.

## Figures and Tables

**Table 1:** The optimal gene sets identified for SKCM and LUAD.

SKCM				
*NBN*	*LIG4*	*MLH1*	*RAD50*	*PMS2*
*FANCA*	*MRE11A*	*PMS1*	*MSH3*	
**LUAD**				
*UBE2T*	*MGMT*	*XPC*	*ALKBH3*	*TDG*
*XRCC2*	*CUL5*	*NBN*	*FANCC*	*BARD1*
*ERCC4*	*MSH2*	*XRCC4*	*UNG*	

## Data Availability

Publicly available datasets were analyzed in this study. This data can be found here: https://gdc.cancer.gov/about-data/publications/mc3-2017 (accessed on 1 February 2021).

## Supplementary Materials

The following are available online at https://www.mdpi.com/article/10.3390/biology10060528/s1, Figure S1: Schematic of the stepwise selection method for discovering the potential optimal gene set, Figure S2: The association between TMB and mutation status of 276 DDR genes in 33 cancer types and pan-cancer, Figure S3: Empirical distribution of effect sizes from the 10,000 randomly sampled non-DDR gene sets, Figure S4: Empirical distribution of effect sizes from the 10,000 randomly sampled remaining DDR gene sets, Figure S5: Cancer-type specific gene set of colon adenocarcinoma (COAD) and its performance as a potential biomarker for stratifying patients with high TMB, Figure S6: Figure S6. Comparison between the identified DDR gene sets and FoundationOne CDx panel.
