# Supplementary material for: DNA Damage Repair Gene Set as a Potential Biomarker for Stratifying Patients with High Tumor Mutational Burden
Source: Biology (Basel). 2021 Jun 14;10(6):528. doi: 10.3390/biology10060528 (PMC8231881; doi:10.3390/biology10060528)
Supplement: Supplementary file 1 [file biology-10-00528-s001.zip › biology-1236566-suppl.pdf]

biology

Supplementary Materials

DNA Damage Repair Gene Set as a Potential Biomarker for Stratifying Patients with High Tumor Mutational Burden

To-Yuan Chiu, Ryan Weihsiang Lin, Chien-Jung Huang, Da-Wei Yeh, and Yu-Chao Wang

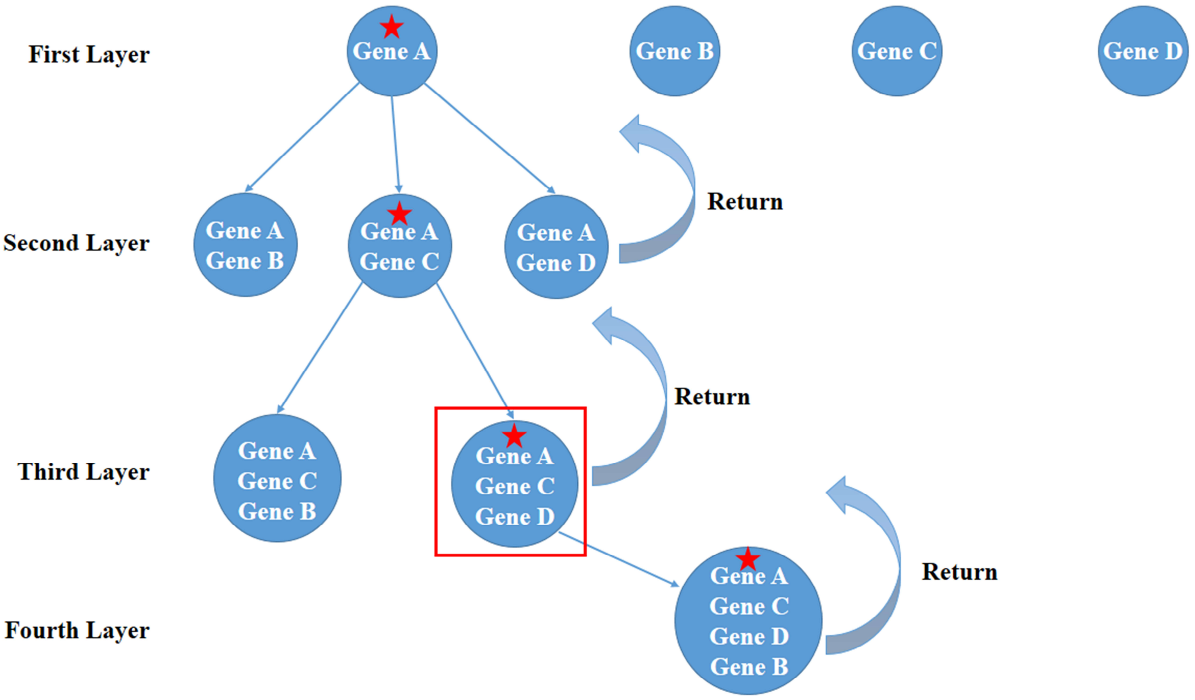

Figure 1. Schematic of the stepwise selection method for discovering the potential optimal gene set. Suppose we have four candidate genes (gene A, gene B, gene C, and gene D) selected. First, we calculated the effect sizes of four candidate genes, respectively. Subsequently, each effect size was transformed into standard score that we could make a comparison of different combinations of gene sets. If the standard score of Gene A was the maximum among these four, Gene A would be selected as the optimal gene set in the first layer. Next, we ran into second layer to add on other genes (Gene B, Gene C, or Gene D) as a subset based on Gene A. Similarly, the effect size and standard score of each subset were calculated. If in this case, a subset with gene A and gene C had the largest standard score, it would be selected as the optimal gene set in the second layer. Then, we used the combination mechanism to select a gene (gene A or gene C) of the current optimal subset (gene A and gene C) and calculated the effect size and its corresponding standard score to search more subsets. If we found the stdscore of the upper layer (first layer) was higher than this layer, we would backtrack. On the contrary, if we found the standard score of the upper layer (first layer) was lower than this layer, we would run into next layer. The procedure would be terminated after one of two conditions was satisfied. The first was that the final layer was encountered. Another was that the optimal gene set still not altered after we dug into five layers. Here, in this example, the gene set with Gene A, Gene C, and Gene D (red rectangle) is identified as the gene set whose mutation status is associated with high TMB.

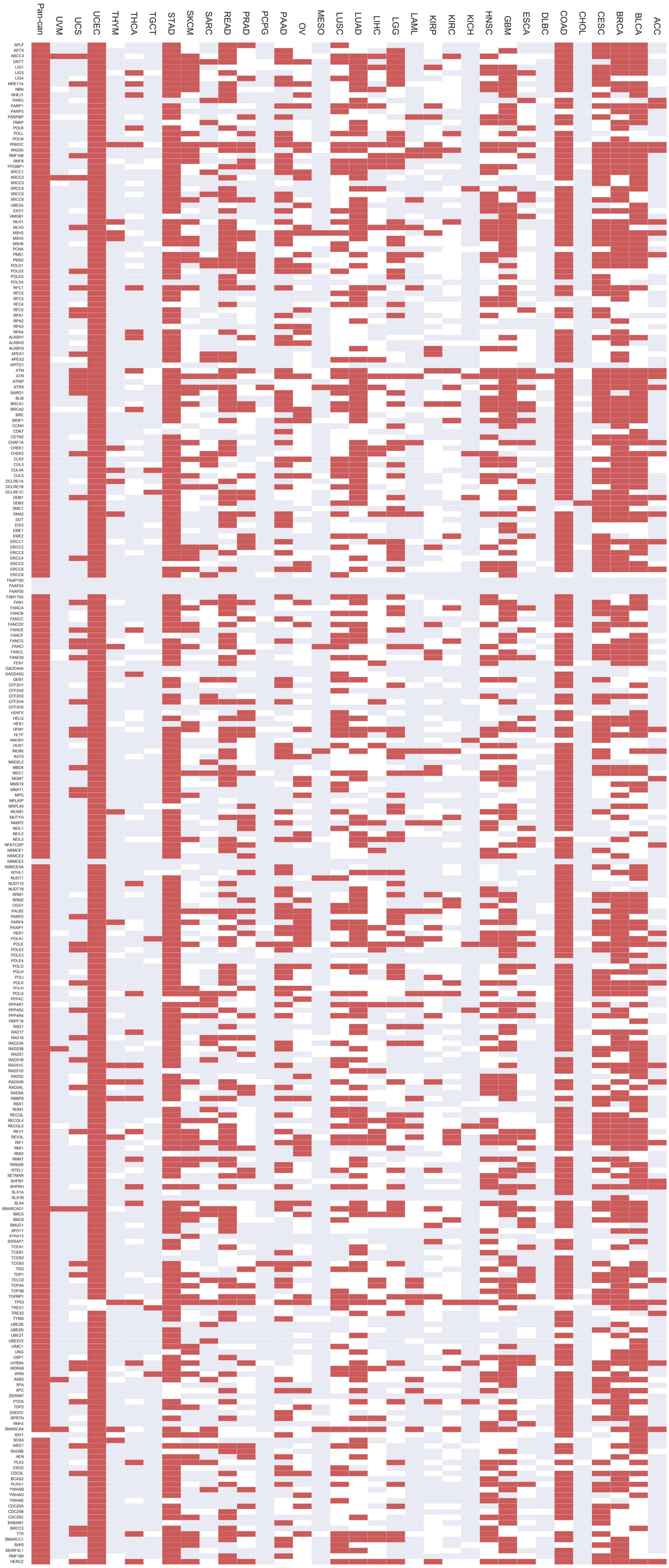
Pan-can
UVM
UCS
UCEC
THYM
THCA
TGCT
STAD
SKCM
SARC
READ
PRAD
PCPG
PAAD
OV
MESO
LUSC
LUAD
LIHC
LGG
LAML
KIRP
KIRC
KICH
HNSC
GBM
ESCA
DLBC
COAD
CHOL
CESC
BRCA
BLCA
ACC

Figure S2. The association between TMB and mutation status of 276 DDR genes in 33 cancer types and pan-cancer. The x-axis represents each of 276 DDR genes. The y-axis represents each of 33 cancer types and pan-cancer. The density of the significant DDR genes in the heatmap is approximately 0.5671.

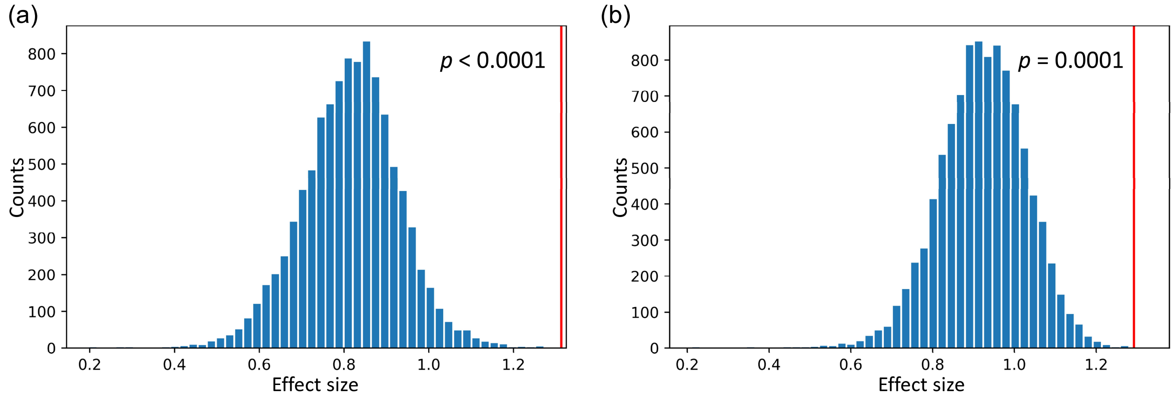

Figure S3. Empirical distribution of effect sizes from the 10,000 randomly sampled non-DDR gene sets: (**a**) SKCM and (**b**) LUAD. Red lines indicate the effect sizes of the identified cancer-specific gene sets. The empirical p-values are shown in the figure.

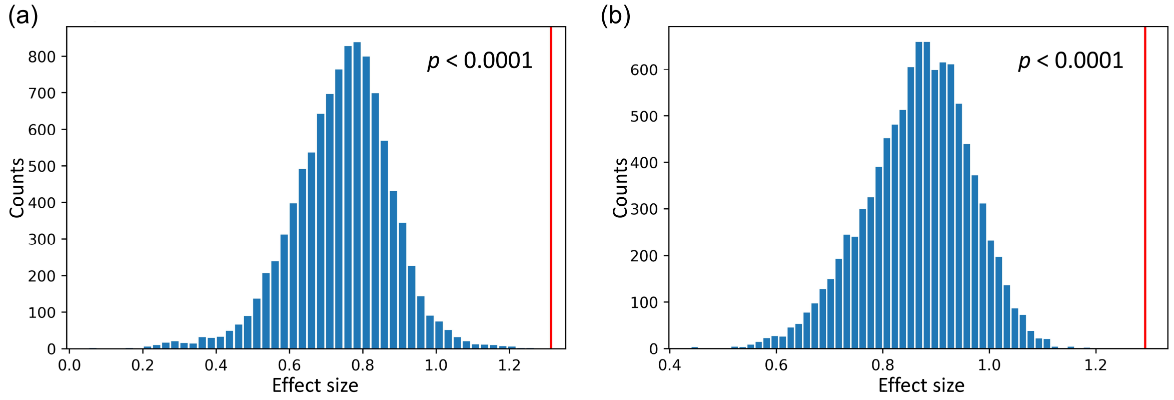

Figure S4. Empirical distribution of effect sizes from the 10,000 randomly sampled remaining DDR gene sets: (**a**) SKCM and (**b**) LUAD. Red lines indicate the effect sizes of the identified cancer-specific gene sets. The empirical p-values are shown in the figure.

(a)

COAD			
ALKBH2	*ALKBH3*	*APEX1*	*POLL*
RNMT	*XPC*	*ERCC5*	*MGMT*
ERCC1	*POLE3*	*FEN1*	*MUS81*
TOP3A	*FANCB*		

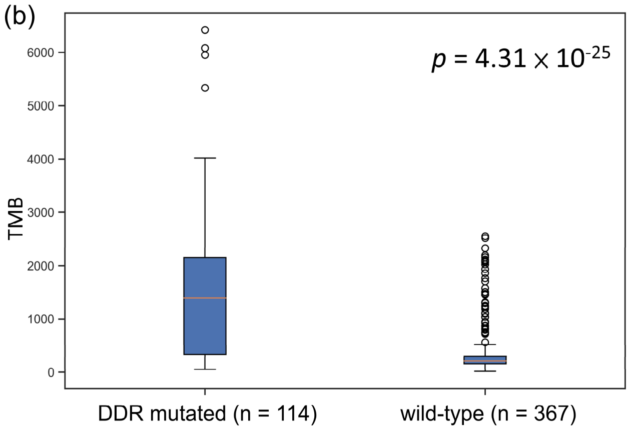

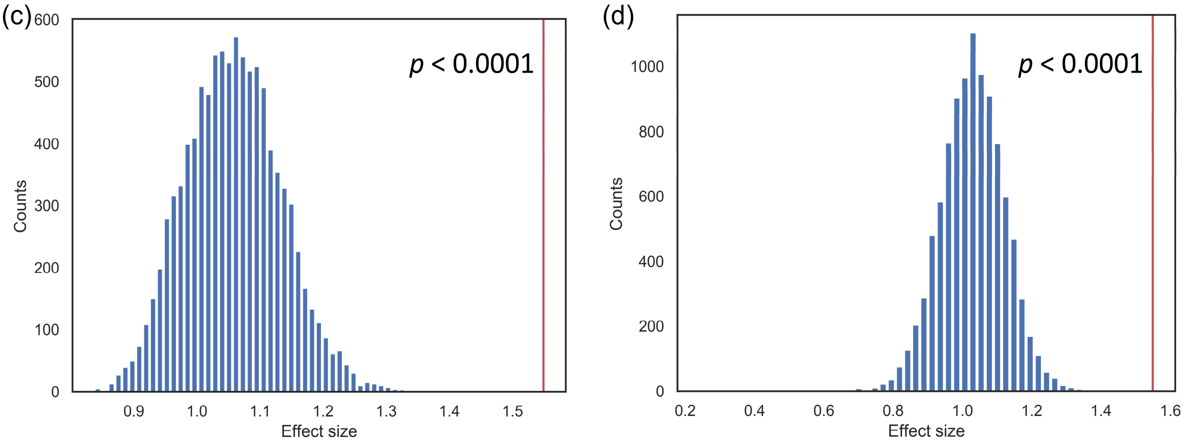

Figure S5. Cancer-type specific gene set of colon adenocarcinoma (COAD) and its performance as a potential biomarker for stratifying patients with high TMB: (**a**) cancer-specific gene set of COAD; (**b**) boxplot of TMB distribution in both mutated and wild-type groups for COAD. *p*-value was calculated by the Mann–Whitney U test; (**c**) empirical distribution of effect sizes from the 10,000 randomly sampled remaining DDR gene sets of COAD. Red line indicates the effect size of the identified cancer-specific gene set. The empirical *p*-value is shown in the figure; (**d**) empirical distribution of effect sizes from the 10,000 randomly sampled non-DDR gene sets of COAD. Red line indicates the effect size of the identified cancer-specific gene set. The empirical *p*-value is shown in the figure.

(a)

	FoundationOne CDx	Identified DDR gene sets
SKCM	0.96	1
LUAD	0.87	0.92
COAD	0.48	1

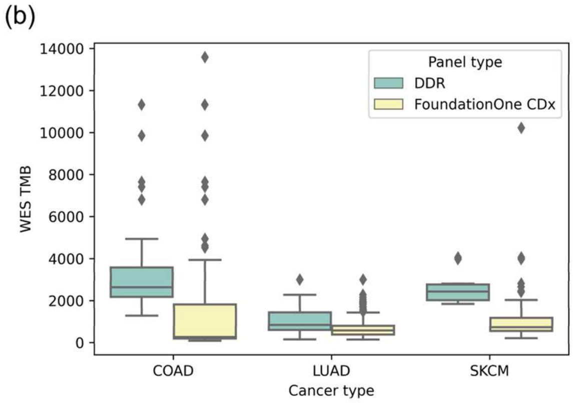

Figure S6. Comparison between the identified DDR gene sets and FoundationOne CDx panel: (**a**) the precision of stratifying TMB-high patients in TCGA SKCM, LUAD, and COAD datasets; (**b**) WES TMB distribution comparison between identified DDR gene sets and FoundationOne CDx panel.
